# Anti-ceramide antibody and sphingosine-1-phosphate as potential biomarkers of unresectable non-small cell lung cancer

**DOI:** 10.3389/pore.2024.1611929

**Published:** 2025-01-06

**Authors:** Lilla Bűdi, Dániel Hammer, Rita Varga, Veronika Müller, Ádám Domonkos Tárnoki, Dávid László Tárnoki, Martina Mészáros, András Bikov, Péter Horváth

**Affiliations:** ^1^ Department of Pulmonology, Semmelweis University, Budapest, Hungary; ^2^ Medical Imaging Centre, Semmelweis University, Budapest, Hungary; ^3^ Wythenshawe Hospital, Manchester University National Health Service Foundation Trust, Manchester Academic Health Science Centre, Manchester, United Kingdom; ^4^ Division of Infection, Immunity and Respiratory Medicine, Faculty of Biology, Medicine and Health, The University of Manchester, Manchester, United Kingdom

**Keywords:** S1P, anti-ceramide antibody, NSCLC, biomarkers, lung cancer

## Abstract

**Objectives:**

Spingosine-1-phosphate (S1P) and ceramides are bioactive sphingolipids that influence cancer cell fate. Anti-ceramide antibodies might inhibit the effects of ceramide. The aim of this study was to assess the potential role of circulating S1P and anti-ceramide antibody as biomarkers in non-small cell lung cancer (NSCLC).

**Methods:**

We recruited 66 subjects (34 controls and 32 patients with NSCLC). Patient history and clinical variables were taken from all participants. Venous blood samples were collected to evaluate plasma biomarkers. If bronchoscopy was performed, bronchial washing fluid (BWF) was also analyzed. We measured the levels of S1P and anti-ceramide antibody with ELISA.

**Results:**

S1P levels were significantly higher in the NSCLC group (3770.99 ± 762.29 ng/mL vs. 366.53 ± 249.38 ng/mL, patients with NSCLC vs. controls, respectively, *p* < 0.001). Anti-ceramide antibody levels were significantly elevated in the NSCLC group (278.70 ± 19.26 ng/mL vs. 178.60 ± 18 ng/mL, patients with NSCLC vs. controls, respectively, *p* = 0.007). Age or BMI had no significant effect on anti-ceramide antibody or S1P levels. BWF samples had higher levels of anti-ceramide antibody (155.29 ± 27.58 ng/mL vs. 105.87 ± 9.99 ng/mL, patients with NSCLC vs. controls, respectively, *p* < 0.001). Overall survival (OS) was 13.36 months. OS was not affected by anti-ceramide antibody or S1P levels.

**Conclusion:**

Higher levels of S1P and anti-ceramide antibody were associated with active cancer. These results suggest that sphingolipid alterations might be important features of NSCLC.

## Introduction

Cancer is the second leading cause of death worldwide, contributing significantly to the global burden of disease. The incidence and mortality rates are rapidly increasing, with lung cancer as the major cause of cancer death [1]. In recent decades, the field of cancer therapy research and clinical implementation has undergone tremendous progress. New therapeutic developments have significantly improved survival rates and quality of life for cancer patients. Currently, innovative strategies such as cell therapies, anti-tumor vaccines and biotechnological drugs are being investigated that shift the focus from a tumor-type towards a gene-centered approach. Biomarker profiling and a better understanding of the complexity of tumor biology are transforming cancer care, leading to more personalized and effective medicine [2–4]. Both genetic/epigenetic alterations in tumor cells and the interaction between tumor cells and the surrounding microenvironment play a crucial role in the tumor formation process. The tumor microenvironment (TME) consists of, among others, tumor cells, tumor stromal cells, immune cells, surrounding blood vessels and the non-cellular components of extracellular matrix [4, 5]. Recent advances in tumor biology have highlighted the importance of understanding the interactions of the TME and their impact on tumor progression and metastasis, potentially leading to development of novel therapeutic strategies. One emerging area of research includes sphingolipids, lipid mediators that have been shown to play pivotal roles in cancer biology [6–8]. Sphingolipid metabolism is a very delicate system that regulates several biological processes, including stress mechanism and inflammation. There is a balance between pro-apoptotic and pro-proliferative bioactive sphingolipids, known as sphingolipid rheostat, which is essential for maintaining normal cell function [9]. Sphingosine-1-phosphate (S1P) and ceramide are important bioactive sphingolipid mediators that influence cancer cell fate. S1P is intracellularly produced in cancer cells and non-cancer stromal cells from sphingosine by two sphingosine kinases (sphingosine kinase 1 (SphK1) and 2 (SphK2)) and then exported to the TME. S1P interacts with cancer and non-cancer cells and other non-cellular components in the TME, to promote cell survival, proliferation, migration, angiogenesis and immune responsiveness, and thus cancer progression [10]. Ceramide is considered to be the central hub of sphingolipid metabolism, as a precursor of all major sphingolipids, including S1P. Ceramide is generated by three major pathways: *de novo* synthesis, the degradation of complex sphingolipids and the recycling of long chain bases [11, 12]. Ceramide acts as a tumor-suppressor lipid, inducing growth arrest, senescence and apoptosis and contributing to stress response [13]. The role of sphingolipid metabolism in cancer pathogenesis, and its potential clinical use in anti-cancer therapy and as a biomarker have been studied *in vitro* and *in vivo* in different cancer types [14–16]. Antibodies against sphingolipid molecules are also under investigation in various diseases. Levels of anti-ceramide antibody in patients with leprosy have been shown to be associated with nerve affection [17]. In another study, anti-ceramide antibody prevented gastrointestinal radiation syndrome in mice by blocking the pro-apoptotic effect of ceramide [18]. Murine anti S1P monoclonal antibody sphingomab inhibited tumor growth in murine renal cell carcinoma xenograft models, both in treatment naive setting and in sunitinib-resistant tumors [19]. The role of anti-ceramide antibody in lung cancer has not been investigated previously. On the basis of previous studies, we hypothesized that anti-ceramide antibody might counteract ceramide-mediated signalling by binding to ceramide in the cell membrane and changing the configuration of receptors in the ceramide rafts.

The aim of this study was to assess the potential role of circulating S1P and anti-ceramide antibody as biomarkers in non-small cell lung carcinoma (NSCLC).

## Materials and methods

### Study subjects and design

The study was cross-sectional in design. Thirty-four control subjects were included, all with a negative history of malignancy. Venous blood samples were taken for biomarker and C-reactive protein (CRP) measurements. We enrolled thirty-two patients with stage III-IV NSCLC who were diagnosed at the Department of Pulmonology, Semmelweis University between 7 October 2021 and 15 August 2022. These patients were referred to our Clinic because of suspected pulmonary malignancy on contrast enhanced chest CT scan. Bronchoscopy was performed as part of the diagnostic evaluation. In case of endobronchial lesions, endobronchial biopsy with biopsy forceps was performed. In patients with peripheral lesions, we performed transbronchial forceps biopsy under fluoroscopic guidance. In cases with mediastinal lymphadenopathy, conventional transbronchial needle aspiration (cTBNA) was also performed. Bronchial washing fluid (BWF) was obtained during the procedure for sphingolipid measurements. BWF was obtained by instilling 20 mL of saline into the affected lobar bronchus. The fluid was then aspirated into a container connected to the suction port of the bronchoscope. Two patients did not have bronchoscopy and were diagnosed on the basis of cytological examination of pleural effusion. On the day of diagnostic bronchoscopy, a complete history was taken, and venous blood samples were collected for plasma sphingolipid measurements and serum CRP levels. The diagnosis of NSCLC was based on histological, and where appropriate, cytological assessment of samples by experienced pathologists at the Department of Pathology, Semmelweis University. Independently of our present study, immunohistochemical (IHC) staining and comprehensive molecular genetic analysis were also performed as part of the routine diagnostic workup at the Department of Pathology, Semmelweis University to support therapeutic decision making. For staging purposes, all patients had brain imaging (contrast enhanced CT or MRI) and either PET-CT scan or contrast enhanced chest and abdominal CT scans. The stage of NSCLC was defined according to the 8th Edition of the UICC TNM classification of Malignant Tumors [20]. None of the patients were eligible for surgical resection.

For BWF measurements we examined the samples of 20 subjects from the NSCLC group. Controls for BWF sphingolipid measurements (n = 3) were patients who underwent bronchoscopy for incidental CT findings of pulmonary nodules and after thorough pulmonary evaluation, were diagnosed with nonmalignant pulmonary disease and had no known current or previous malignancy. As bronchoscopy is only performed with a clear indication, the collection of BWF samples from control subjects was extremely limited. All procedures involving human participants were in accordance with the ethical standards of the institutional and/or national research committee and with the 1964 Helsinki declaration and its later amendments or comparable ethical standards. The study was approved by the local Ethics Committee (Semmelweis University, TUKEB 215/2021 and TUKEB 30/2014), and informed consent was obtained from all participating volunteers.

### Clinical characteristics of study population

Clinical data and subject characteristics are summarized in Table 1. Patients with NSCLC were older, had a higher prevalence of smoking, chronic obstructive pulmonary disease (COPD) and hypertension, and had higher levels of circulating CRP (all *p* < 0.05). S1P and anti-ceramide antibody levels were not affected by the prevalence of COPD in the NSCLC group (Supplementary Material 1). There was no significant difference in sex distribution between the NSCLC and the control group.

**TABLE 1:** Clinical characteristics of controls and patients with NSCLC.

	Control (n = 34)	NSCLC (n = 32)	*p*-value
Age, years	46 [20–74]	65.5 [45–92]	<0.001
Male	11 (32.35)	15 (46.88)	0.34
BMI, kg/m^2^	24.14 [17.21–41.02]	24.35 [17.9–33.56]	0.46
Hypertension	9 (26.47)	25 (78.13)	<0.001
Diabetes	2 (5.88)	6 (18.75)	0.22
COPD	2 (5.88)	12 (37.50)	0.004
Asthma	2 (5.88)	1 (3.13)	1
Smoking	1 (2.94)	24 (75.00)	<0.001
CRP, mg/L	1.17 [0.22–5.59]	12.0 [0.4–271]	<0.001

Data are median [range] or numbers (%). NSCLC, non-small cell lung cancer; BMI, body mass index; COPD, chronic obstructive pulmonary disease; CRP, C-reactive protein.

### Oncological characteristics in the NSCLC group

Oncological data are summarized in Table 2. On the basis of histological assessment, 71.87% and 28.12% of the samples were characterized as adenocarcinoma and squamous cell carcinoma (SCC), respectively. PD-L1 IHC staining was positive (TPS≥1%) in 8 cases (88.89%) of SCC and 15 cases (65.22%) of adenocarcinoma. KRAS mutations were detected in 14 cases (60.87%), ALK fusion in 2 cases (8.70%), EGFR mutation in 1 case (4.34%) and ROS1 translocation in 1 case (4.34%) of adenocarcinoma samples. Molecular genetic analysis is not available in 2 cases of adenocarcinoma: one patient deceased shortly after histological sampling and one patient did not return to our facility, no further data is available on them in our database. 12.5%, 12.5%, and 75% of the patients had stage IIIA, stage IIIB and stage IV NSCLC, respectively. Median OS was 13.36 months from the date of diagnosis.

**TABLE 2:** Summary of oncological data of NSCLC patients (n = 32).

Stage (%)	
IIIA	4 (12.5)
IIIB	4 (12.5)
IV	24 (75)
ECOG PS (%)	
0–1	23 (71.88)
2	8 (25)
3–4	1 (3.12)
Histology (%)	
SCC	9 (28.13)
Adenocarcinoma	23 (71.87)
PDL1 IHC (%)	
SCC PDL1	
TPS <1%	1 (11.11)
TPS 1%–50%	6 (66.67)
TPS >50%	2 (22.22)
Adenocarcinoma PD-L1	
TPS <1%	6 (26.09)
TPS 1%–50%	11 (47.83)
TPS >50%	4 (17.39)
NA	2 (8.69)
Molecular oncological analysis – Adenocarcinoma (%)	
ALK fusion	2 (8.70)
KRAS mutation	14 (60.87)
p.G12C	12 (85.71)
p.G12V	2 (14.29)
EGFR mutation (exon 18, p.G719A)	1 (4.35)
ROS1 translocation	1 (4.35)
No targetable mutation detected	3 (13.04)
NA	2 (8.69)

Data are presented in numbers (%). NSCLC, non-small cell lung cancer; SCC, squamous cell carcinoma; ECOG PS, Eastern Cooperative Oncology Group Performance Status Scale; IHC, immunohistochemistry; PD-L1, programmed cell death ligand 1; ALK, anaplastic lymphoma kinase; EGFR, epidermal growth factor receptor; KRAS, Kirsten rat sarcoma viral oncogene; ROS1, c-ros oncogene 1; BRAF- v-raf murine sarcoma viral oncogene homolog B1; NA, not available.

### S1P and anti-ceramide antibody measurements

Venous blood samples were collected in EDTA tubes. Within 2 h of sample collection, EDTA tubes were centrifuged at 1,500 g. We separated plasma from the cellular content. Plasma was immediately stored at −80°C until analysis. BWF was collected in tracheal suction sets and stored at −80°C. Commercially available ELISA kits were used for measurements of plasma S1P and anti-ceramide antibody levels and BWF anti-ceramide antibody levels [MyBioSource Inc., San Diego, CA, United States; Human S1P ELISA kit (MBS2516132) and Human Anti-Ceramide Antibody ELISA kit (MBS3804520)]. Measurements were performed in duplicates according to the manufacturer’s instructions and mean concentrations were used as inputs for analysis. Samples were used at a fourfold dilution for ceramide antibody measurement and at a tenfold dilution for S1P measurements.

### Statistical analysis

Statistical analysis was performed using the R statistical software program v. 4.1.3 (R Statistical Foundation, Vienna, Austria). Normality of data was assessed using the Shapiro-Wilk test. Anti-ceramide antibody and S1P levels were compared between the two groups with linear regression. We adjusted the values for age and BMI. Anti-ceramide antibodies showed no correlation with age, sex or BMI. S1P showed a moderate positive correlation with age (rho = 0.50). Categorical variables were compared with the chi-squared test. Data are expressed as median [range] or mean ± SD. *P* values of <0.05 were considered significant. The sample size was estimated to detect differences of at least 70% of the standard deviation (0.70 effect size) in either anti-ceramide antibody or S1P levels between the two groups with a power of 0.80 and α error probability of 0.05 [21]. Survival analysis was performed with the survival package of R, Kaplan Meier plots were drawn with the survminer package. We used ROC analysis to calculate the cutoff value of anti-ceramide antibodies (rocit and OptimalCutpoints packages of R, Supplementary Material 2). Using this cutoff, we classified patients as having high or low anti-ceramide antibody levels. In case of S1P we used the median value as cutoff in determining high and low levels. Boxplots were drawn using SPSS 29.0.1.0.

## Results

### Comparison of plasma S1P levels between the NSCLC and control groups

**FIGURE 1:**
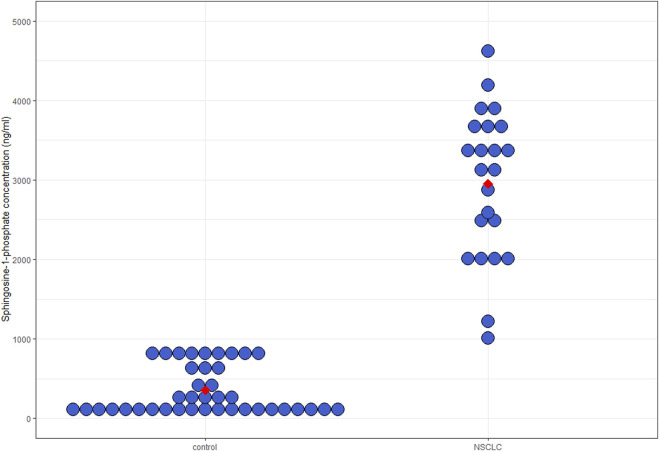
Plasma S1P levels. Median circulating S1P levels were significantly higher in NSCLC patients than in controls (3770.99 ± 762.29 ng/mL vs. 366.53 ± 249.38 ng/mL, patients with NSCLC vs. controls, respectively, *p* < 0.001). S1P, sphingosine-1-phosphate; NSCLC, non-small cell lung cancer.

We detected significantly higher S1P levels in the NSCLC group than in the control group (3770.99 ± 762.29 ng/mL vs. 366.53 ± 249.38 ng/mL, patients with NSCLC vs controls, respectively, *p* < 0.001) (Figure 1). Age or BMI had no significant effect on S1P levels (*p* = 0.13 and 0.27, respectively). We observed no difference between S1P levels in terms of KRAS gene mutation status (3842.79 ng/mL [374.3–4190.4 ng/mL] vs. 3923.53 [3397.8–4349.3 ng/mL], KRAS mutant vs. KRAS wild type, respectively, *p* = 0.37). There was no significant difference regarding PDL1 status either (3691.76 ng/mL [3397.8–4281.4 ng/mL] vs. 3931.16 ng/mL [374.34–4349.33 ng/mL], PDL1 negative vs. PDL1 positive cases, respectively, *p* = 0.89).

### Comparison of plasma anti-ceramide antibody levels between the NSCLC and control groups

**FIGURE 2:**
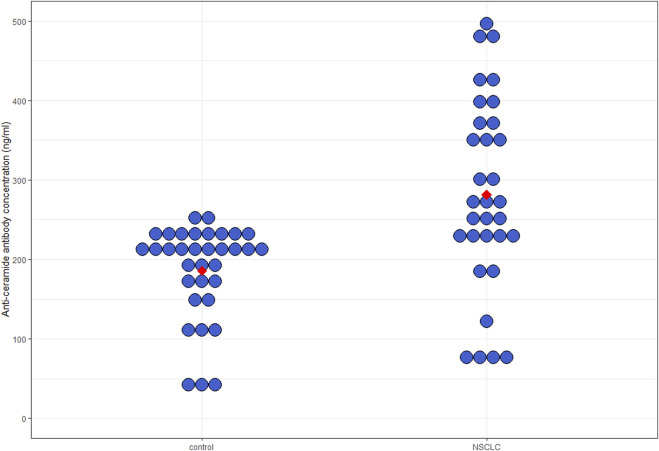
Plasma anti-ceramide antibody levels. Median circulating anti-ceramide antibody levels in patients with NSCLC were significantly elevated compared to controls (278.70 ± 19.26 ng/mL vs. 178.60 ± 18 ng/mL, patients with NSCLC vs. controls, respectively, *p* = 0.007). NSCLC, non-small cell lung cancer.

Anti-ceramide antibody levels were significantly elevated in the NSCLC group (278.70 ± 19.26 ng/mL vs. 178.60 ± 18 ng/mL, patients with NSCLC vs. controls, respectively, *p* = 0.007) (Figure 2). Age or BMI had no significant effect on anti-ceramide antibody levels (*p* = 0.11 and 0.27, respectively). We observed no difference between anti-ceramide antibody levels in terms of KRAS gene mutation status (287.44 ng/mL [71.40–496.20 ng/mL] vs. 256.05 [121.67–403.01 ng/mL], KRAS mutant vs. KRAS wild type, respectively, *p* = 0.79). In terms of PDL1 status, there was no significant difference between PDL1 positive and negative NSCLC either (268.90 ng/mL [71.40–496.20 ng/mL vs. 256.05 ng/mL [76.52–392.71 ng/mL], respectively, *p* = 0.50).

### Comparison of BWF anti-ceramide antibody levels between the NSCLC and control groups

**FIGURE 3:**
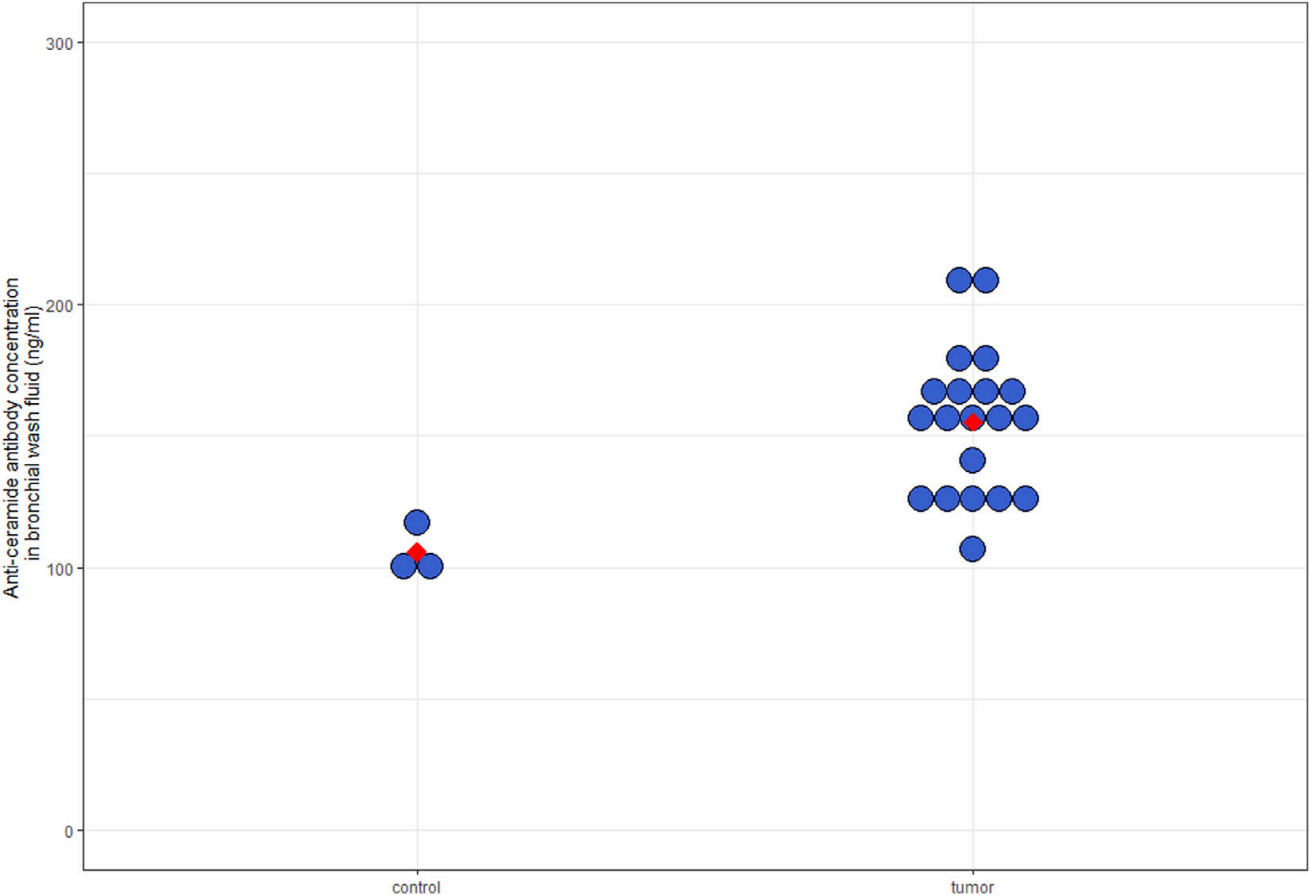
Anti-ceramide antibody levels in BWF samples. Median anti-ceramide antibody levels in BWF samples were significantly higher in NSCLC patients compared to controls (155.29 ng/mL ± 27.58 ng/mL vs. 105.87 ng/mL ± 9.99 ng/mL, patients with NSCLC vs. controls, respectively, *p* < 0.001); however, these results are limited due to small sample size. BWF, bronchial washing fluid; NSCLC, non-small cell lung cancer.

We measured significantly elevated anti-ceramide antibody levels in the NSCLC group (155.29 ng/mL ± 27.58 ng/mL vs. 105.87 ng/mL ± 9.99 ng/mL, patients with NSCLC vs. controls, respectively, *p* < 0.001). However, these results are limited due to the small number of controls (Figure 3).

### Survival analysis in the NSCLC group

**FIGURE 4:**
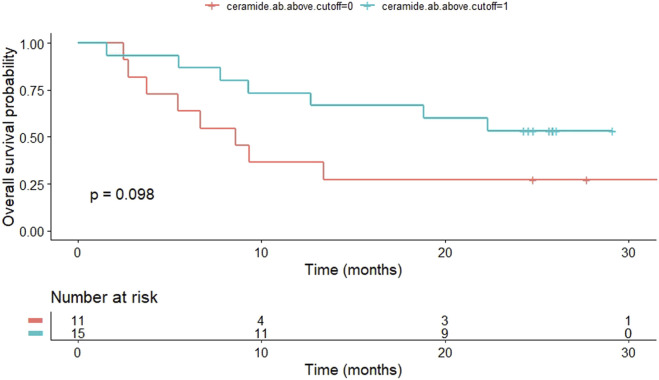
Kaplan-Meier curve shows OS of patients with NSCLC according to anti-ceramide antibody levels. Patients with high levels of anti-ceramide antibody had a tendency for prolonged OS (8.6 months [1.56–29.13 months] vs. 24.3 months [2.5–33.63 months], patients with low vs. high anti-ceramide antibody levels, respectively, *p* = 0.098). Patients were classified as having high anti-ceramide antibody levels if they were higher than 184.5 ng/mL. OS, overall survival; NSCLC, non-small cell lung cancer.

**FIGURE 5:**
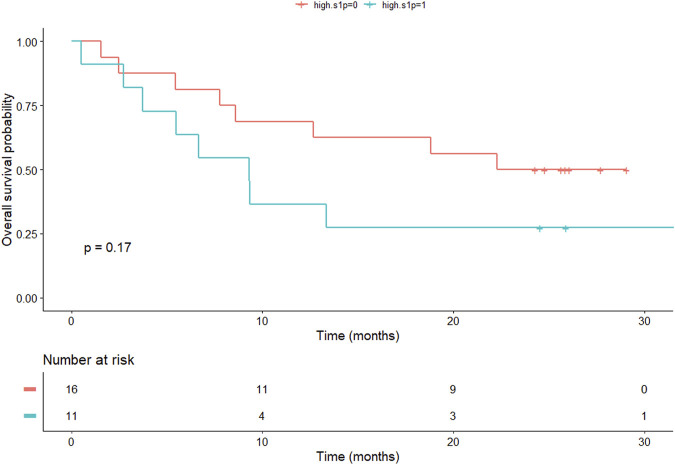
Kaplan Meier curve shows OS of patients with NSCLC according to S1P levels. We found no significant difference in OS (9.3 months vs. 22.3 months in the low S1P and high S1P groups, respectively). OS, overall survival; NSCLC, non-small cell lung cancer; S1P, sphingosine-1-phosphate.

Overall survival (OS) was a secondary end point in our study. Six patients were lost to follow-up and therefore excluded from the survival analysis. Patients with high levels of anti-ceramide antibody had a tendency for prolonged OS (8.6 months [1.56–29.13 months] vs. 24.3 months [2.5–33.63 months], patients with low vs. high anti-ceramide antibody levels, respectively, *p* = 0.098). We used a cutoff of 184.5 ng/mL based on ROC analysis (Figure 4). In case of S1P levels, we found no significant difference in OS. We used the median value of S1P levels (3926.52 ng/mL) as cutoff (Figure 5). We must emphasize however, that the survival data is exploratory and preliminary due to the small patient cohort.

## Discussion

We investigated the possible role of sphingolipid regulation in NSCLC. Our results showed an association between the upregulation of S1P and anti-ceramide antibody levels in active cancer. Patients in the NSCLC group had a higher prevalence of smoking, hypertension and COPD, and elevated CRP levels compared to controls. No patients had active infection at the time of CRP level measurement. Though COPD is an important factor of lung injury, we found that S1P and anti-ceramide antibody levels were not affected by the prevalence of COPD in the NSCLC group. These differences in cofounding factors between the two groups may be due to the fact that smoking is a strong risk factor for hypertension, COPD and lung cancer [22]. Elevated CRP levels are possibly in association with the systemic cancer-related inflammation, as CRP is a non-specific marker for inflammation [23]. Chronic inflammation is known to underlie many physiological and pathophysiological processes and is strongly associated with cancer. During the inflammatory process, several bioactive molecules are synthesized, including lipid mediators. The role of sphingolipid metabolism in cancer, anti-cancer therapy and the potential routine clinical application is a widely studied topic [24].

The two main bioactive sphingolipids are pro-proliferative S1P and pro-apoptotic ceramide [25]. S1P levels were significantly higher in patients with NSCLC. S1P is a downstream metabolite of ceramide, synthesized intracellularly by Sphk1 and Sphk2 by phosphorylation of sphingosine [26]. Sphingolipid metabolism is regulated by a network of specific and compartmentalized enzymes. In the development of cancer, the dysregulation of this system results in the accumulation of pro-proliferative sphingolipids [27]. S1P is a bioactive sphingolipid involved in multiple signaling pathways and functions as a pro-inflammatory, pro-survival signaling molecule [25]. S1P promotes inflammation and cancer progression both extra- and intracellularly. In addition to acting as a signaling molecule in autocrine, paracrine and intracellular pathways, it has been shown to epigenetically regulate gene transcription in various biological processes [6, 24]. High S1P levels are consistent with previous findings and support the theory that S1P may be a valuable biomarker in cancer. Plasma S1P levels were significantly elevated in ovarian cancer [28] and circulating levels of S1P were higher in patients with hepatocellular carcinoma than in those with liver cirrhosis [29]. Elevated circulating levels of sphingolipids S1P and ceramide were associated with an increased risk of future lung cancer [30]. Alterations in sphingolipid metabolic gene expression B3GNT5 and GAL3ST1 were also identified in patients with NSCLC that reflected into the serum levels of their metabolites lacto/neolacto-series glycosphingolipids and sulfatides, implying their potential roles as biomarkers for NSCLC [31]. In a study similar to ours glycerophospho-N-arachidonoyl ethanolamine (GpAEA) and sphingosine were promising potential biomarkers in NSCLC [32]. To the best of our knowledge, our study is the first to measure S1P levels in diagnosed unresectable and advanced stage NSCLC patients. In terms of the unequivocal difference between patients with NSCLC and controls, S1P may be a sensitive, non-invasive biomarker for cancer diagnosis. As our study was cross-sectional in design, it can only be speculated whether changes in S1P levels during the course of the disease correlate with disease progression. In addition to being a promising biomarker, S1P is being investigated for potential therapeutic roles in a variety of diseases. At present, the only approved indication is the use of S1P receptor (S1PR) modulators in the treatment of relapsing remitting and active secondary multiple sclerosis [33]. Targeting the S1P signaling pathway could lead to possible new anti-cancer therapies. Several agents are under development, including S1P-specific neutralizing antibodies, Sphk inhibitors and functional S1P receptor antagonists [34].

Anti-ceramide antibody levels were also elevated in plasma and BWF samples from patients with NSCLC. Ceramides are a family of lipid molecules that were first identified as important structural components of cell membranes. With the improvement of sphingolipidomics, various functions of ceramide as a bioactive sphingolipid, including its crucial role in cell survival, have been discovered. Ceramide, which promotes cell growth arrest, cell senescence and apoptosis, is a major mediator in cancer pathogenesis, mainly acting as a tumor suppressor [27]. Ceramide has also been shown to induce cell death by autophagy. The main intracellular signaling pathways that contribute to ceramide induced lethal autophagy are the mTOR and bcl pathway [35]. We hypothesized that anti-ceramide antibodies may have a neutralizing effect against ceramide molecules. The results of a former study support our hypothesis, as ceramide antibodies were shown to be protective in radiational gastrointestinal syndrome in mice by binding to ceramide and thus preventing the transmission of an apoptotic signal [18]. We hypothesized that TME may produce anti-ceramide antibodies in order to promote cancer cell survival by inhibiting the pro-apoptotic effects of ceramide. We measured significantly higher levels of anti-ceramide antibody not only in plasma but also in BWF samples in patients with NSCLC. The fact that anti-ceramide antibody levels were elevated in BWF samples, supports our assumption that anti-ceramide antibody may be produced directly by the tumor and its microenvironment. Further studies are needed to confirm our hypothesis, for instance by detecting anti-ceramide antibodies with IHC directly in tumor samples. Ceramide inducers and analogues are currently under investigation as potential anticancer drugs alone or in combination with chemotherapy and immunotherapy [34, 36]. Ceramide synthesis is also induced by many chemotherapeutic drugs, either by *de novo* synthesis, or through sphingomyelinases. This contributes to the anti-tumor effect of chemotherapeutic treatment [37]. Pyridinium ceramide has been shown to be effective in inhibiting the growth of head and neck squamous cell carcinoma and pancreatic cell lines [38, 39]. Targeting accumulated survivin by nanoliposomal ceramide resulted in complete remission of natural killer type of aggressive large granular lymphocytic leukemia in a rat model [40].

As a secondary endpoint, we assessed the median OS using the Kaplan-Meier method. The median OS observed in this study was generally consistent with reports in the literature [41, 42]. Anti-ceramide antibody and S1P levels did not significantly affect OS. However, high levels of anti-ceramide antibody levels showed a tendency for prolonged OS, which might prove significant in a statistically more powerful cohort.

This study has some limitations. First, it was designed to detect differences in anti-ceramide antibody and S1P levels between two groups, the sample size might be small to draw conclusions on subgroup analysis and correlations. Also, the small patient cohort is a limitation for excessive survival analysis, survival data are preliminary and exploratory. Another limitation is the difference between the control and NSCLC groups regarding cofounding factors such as smoking status, age and COPD. Since the study was cross-sectional in design, additional investigations are needed to assess the prognostic value of sphingolipid level measurements after completion of first-line therapy to better understand the role of sphingolipids in the pathomechanism of NSCLC.

In conclusion, the evaluation of this complex system has the potential to improve our understanding of the mechanisms of carcinogenesis, cancer cell survival and metastasizing, and to lead to the identification of novel diagnostic and prognostic biomarkers and targets for anti-cancer therapy. Despite advances in anti-cancer therapy, lung cancer remains the leading cause of cancer-related mortality, mainly due to advanced stage at diagnosis. Identifying reliable biomarkers of NSCLC and potential targets for therapy may improve the outcomes for patients with NSCLC.

## Data Availability

The original contributions presented in the study are included in the article/Supplementary Material, further inquiries can be directed to the corresponding author.
