# Supplementary material for: Anti-ceramide antibody and sphingosine-1-phosphate as potential biomarkers of unresectable non-small cell lung cancer
Source: Pathol Oncol Res. 2025 Jan 6;30:1611929. doi: 10.3389/pore.2024.1611929 (PMC11742942; doi:10.3389/pore.2024.1611929)
Supplement: Supplementary file 1 [file DataSheet2.PDF]

Subgroup analysis of NSCLC patients

2024-11-03

Analysis of the effect of COPD on biomarker levels

- Anti-Ceramide antibody (t-test comparing COPD patients with non-COPD patients in the NSCLC subgroup)

```
t.test(data_nsclc[data_nsclc$COPD == 0,]$cerab, data_nsclc[data_nsclc$COPD ==
1,]$cerab)

##
##  Welch Two Sample t-test
##
## data:  data_nsclc[data_nsclc$COPD == 0, ]$cerab and
data_nsclc[data_nsclc$COPD == 1, ]$cerab
## t = 0.91868, df = 27.3, p-value = 0.3663
## alternative hypothesis: true difference in means is not equal to 0
## 95 percent confidence interval:
##  -47.72553 125.18290
## sample estimates:
## mean of x mean of y
##  295.9332  257.2045
```

- Sphingosine-1-phosphate (t-test comparing COPD patients with non-COPD patients in the NSCLC subgroup)

```
t.test(data_nsclc[data_nsclc$COPD == 0,]$s1p.level,
data_nsclc[data_nsclc$COPD == 1,]$s1p.level)

##
##  Welch Two Sample t-test
##
## data:  data_nsclc[data_nsclc$COPD == 0, ]$s1p.level and
data_nsclc[data_nsclc$COPD == 1, ]$s1p.level
## t = -1.631, df = 16.658, p-value = 0.1216
## alternative hypothesis: true difference in means is not equal to 0
## 95 percent confidence interval:
##  -1016.7853   130.9231
## sample estimates:
## mean of x mean of y
##  3576.101  4019.032
```

There is no statistically significant difference in the level of either biomarkers with regards to COPD status.
