# Supplementary material for: Anti-ceramide antibody and sphingosine-1-phosphate as potential biomarkers of unresectable non-small cell lung cancer
Source: Pathol Oncol Res. 2025 Jan 6;30:1611929. doi: 10.3389/pore.2024.1611929 (PMC11742942; doi:10.3389/pore.2024.1611929)
Supplement: Supplementary file 2 [file DataSheet1.PDF]

ROC analysis

2024-11-03

ROC analysis of S1P in our cohort

```
roc.s1p <- rocit(data$s1p.levels, data$cancer)

## Warning in rocit(data$s1p.levels, data$cancer): NA(s) in score and/or
class,
## removed from the data.
```

ROC plot of S1P sensitivity and 1-Specificity:

```
plot(roc.s1p)
```

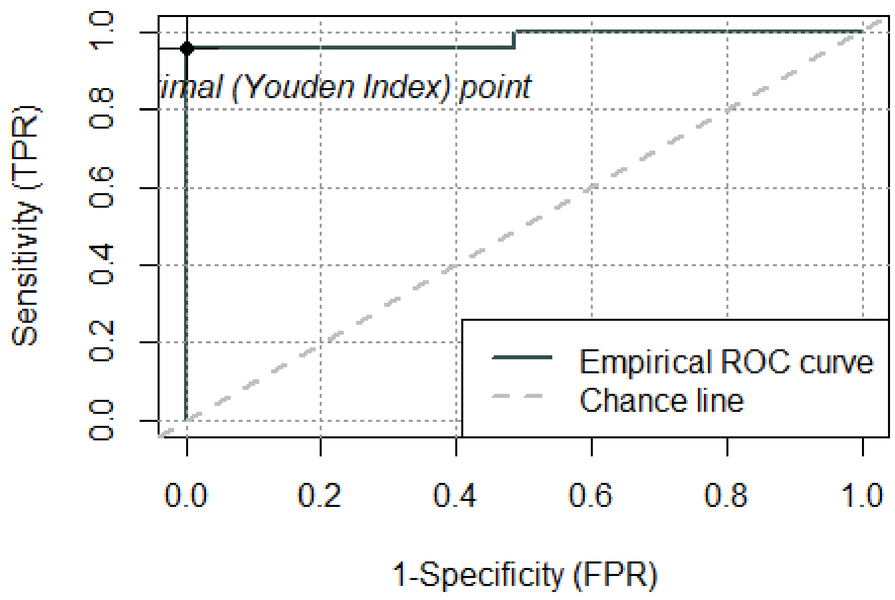

Table of TPR, FPR and cutoff values:

```
cbind(roc.s1p$TPR, roc.s1p$FPR, roc.s1p$Cutoff)

##       [,1]       [,2]    [,3]
##  [1,] 0.00 0.00000000     Inf
##  [2,] 0.04 0.00000000 4532.19
##  [3,] 0.08 0.00000000 4349.33
##  [4,] 0.12 0.00000000 4290.86
##  [5,] 0.16 0.00000000 4281.45
```

```
##  [6,] 0.20 0.00000000 4190.45
##  [7,] 0.24 0.00000000 4121.02
##  [8,] 0.28 0.00000000 4063.96
##  [9,] 0.32 0.00000000 4035.94
## [10,] 0.36 0.00000000 4028.30
## [11,] 0.40 0.00000000 3968.78
## [12,] 0.44 0.00000000 3955.81
## [13,] 0.48 0.00000000 3931.16
## [14,] 0.52 0.00000000 3925.62
## [15,] 0.56 0.00000000 3921.44
## [16,] 0.60 0.00000000 3754.43
## [17,] 0.64 0.00000000 3710.98
## [18,] 0.68 0.00000000 3702.57
## [19,] 0.72 0.00000000 3691.76
## [20,] 0.76 0.00000000 3688.27
## [21,] 0.80 0.00000000 3623.29
## [22,] 0.84 0.00000000 3605.63
## [23,] 0.88 0.00000000 3602.99
## [24,] 0.92 0.00000000 3526.30
## [25,] 0.96 0.00000000 3397.89
## [26,] 0.96 0.02564103  969.75
## [27,] 0.96 0.05128205  903.51
## [28,] 0.96 0.07692308  812.09
## [29,] 0.96 0.10256410  669.11
## [30,] 0.96 0.12820513  663.28
## [31,] 0.96 0.15384615  646.08
## [32,] 0.96 0.17948718  635.75
## [33,] 0.96 0.20512821  607.06
## [34,] 0.96 0.23076923  568.95
## [35,] 0.96 0.25641026  499.83
## [36,] 0.96 0.28205128  483.57
## [37,] 0.96 0.30769231  464.99
## [38,] 0.96 0.33333333  451.50
## [39,] 0.96 0.35897436  435.38
## [40,] 0.96 0.38461538  424.83
## [41,] 0.96 0.41025641  424.05
## [42,] 0.96 0.43589744  407.30
## [43,] 0.96 0.46153846  406.01
## [44,] 0.96 0.48717949  380.61
## [45,] 1.00 0.48717949  374.34
## [46,] 1.00 0.51282051  345.50
## [47,] 1.00 0.53846154  316.00
## [48,] 1.00 0.56410256  302.84
## [49,] 1.00 0.58974359  292.62
## [50,] 1.00 0.61538462  264.03
## [51,] 1.00 0.64102564  257.98
## [52,] 1.00 0.66666667  239.03
## [53,] 1.00 0.69230769  236.33
## [54,] 1.00 0.71794872  223.64
## [55,] 1.00 0.74358974  223.63
```

```
## [56,] 1.00 0.76923077  218.43
## [57,] 1.00 0.79487179  149.37
## [58,] 1.00 0.82051282  148.04
## [59,] 1.00 0.84615385  101.65
## [60,] 1.00 0.87179487   75.64
## [61,] 1.00 0.89743590   46.54
## [62,] 1.00 1.00000000    0.00
```

```
summary(optimal.cutpoints(s1p.levels~cancer, tag.healthy  = 0, methods =
"ValueDLR.Negative", data = data))
```

```
## Warning: There is no cutpoint that yields the exact Diagnostic Negative
##  Likelihood Ratio designated. The cutpoint having the closest value to the
##  designated Diagnostic Negative Likelihood Ratio has therefore been
selected.
```

```
##
## Call:
## optimal.cutpoints.formula(X = s1p.levels ~ cancer, tag.healthy = 0,
##     methods = "ValueDLR.Negative", data = data)
##
## Area under the ROC curve (AUC):  0.981 (0.942, 1.019)
##
## CRITERION: ValueDLR.Negative
## Number of optimal cutoffs: 2
##
##                  Estimate
## cutoff       3925.6200000
## Se              0.5200000
## Sp              1.0000000
## PPV             1.0000000
## NPV             0.7647059
## DLR.Positive          Inf
## DLR.Negative    0.4800000
## FP              0.0000000
## FN             12.0000000
##
##              Estimate
## cutoff        3931.16
## Se               0.48
## Sp               1.00
## PPV              1.00
## NPV              0.75
## DLR.Positive      Inf
## DLR.Negative     0.52
## FP               0.00
## FN              13.00
```

ROC analysis of anti-ceramide antibody levels in our cohort

```
roc.cerab <- rocit(data$cerab, data$cancer)
```

ROC plot of anti-ceramide antibody sensitivity and 1-Specificity:

```
plot(roc.cerab)
```

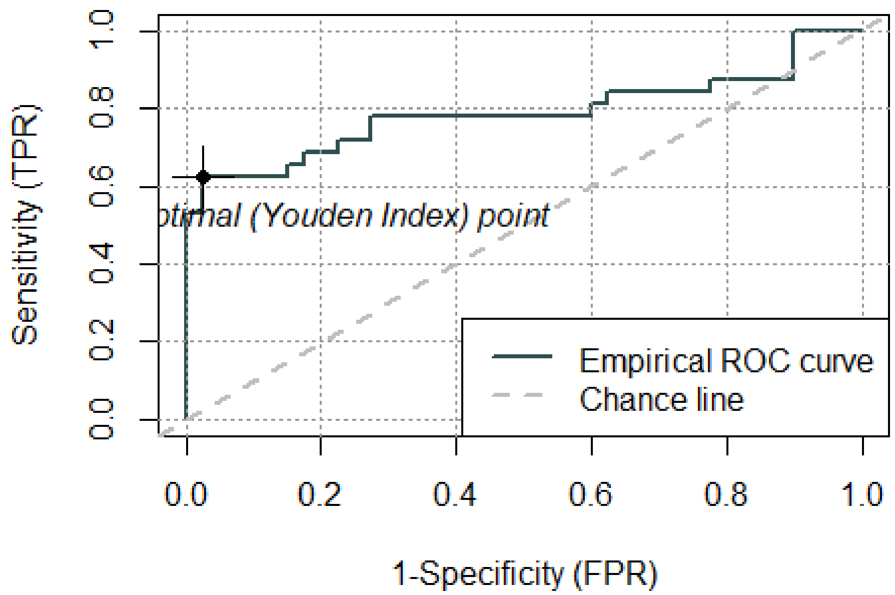

Table of TPR, FPR and cutoff values:

```
cut.values <- as.data.frame(cbind(roc.cerab$TPR, roc.cerab$FPR,
roc.cerab$Cutoff))
names(cut.values) <- c("TPR", "FPR", "Cutoff")
cut.values
```

```
##        TPR   FPR    Cutoff
## 1  0.00000 0.000       Inf
## 2  0.03125 0.000 496.20710
## 3  0.06250 0.000 483.14269
## 4  0.09375 0.000 477.30283
## 5  0.12500 0.000 432.02890
## 6  0.15625 0.000 418.90427
## 7  0.18750 0.000 403.01023
## 8  0.21875 0.000 392.71523
## 9  0.25000 0.000 378.74774
## 10 0.28125 0.000 364.35882
## 11 0.31250 0.000 357.61589
## 12 0.34375 0.000 355.80975
## 13 0.37500 0.000 342.20349
## 14 0.40625 0.000 304.63576
## 15 0.43750 0.000 296.02649
## 16 0.46875 0.000 276.88140
```

```
## 17 0.50000 0.000 270.25880
## 18 0.53125 0.000 267.54967
## 19 0.53125 0.025 258.82353
## 20 0.56250 0.025 256.05057
## 21 0.59375 0.025 255.26791
## 22 0.62500 0.025 246.05659
## 23 0.62500 0.050 245.58824
## 24 0.62500 0.075 244.11765
## 25 0.62500 0.100 239.70588
## 26 0.62500 0.125 238.23529
## 27 0.62500 0.150 237.50000
## 28 0.65625 0.150 237.02589
## 29 0.65625 0.175 231.61765
## 30 0.68750 0.175 230.64419
## 31 0.68750 0.200 230.14706
## 32 0.68750 0.225 228.67647
## 33 0.71875 0.225 226.61048
## 34 0.71875 0.275 224.26471
## 35 0.75000 0.275 223.35942
## 36 0.78125 0.275 221.37267
## 37 0.78125 0.300 219.85294
## 38 0.78125 0.325 218.38235
## 39 0.78125 0.350 213.23529
## 40 0.78125 0.375 210.29412
## 41 0.78125 0.400 209.55882
## 42 0.78125 0.425 208.08824
## 43 0.78125 0.450 207.35294
## 44 0.78125 0.475 206.61765
## 45 0.78125 0.500 205.88235
## 46 0.78125 0.525 204.41176
## 47 0.78125 0.550 200.73529
## 48 0.78125 0.575 200.00000
## 49 0.78125 0.600 194.85294
## 50 0.81250 0.600 188.32029
## 51 0.81250 0.625 184.55882
## 52 0.84375 0.625 180.79470
## 53 0.84375 0.650 177.20588
## 54 0.84375 0.675 172.05882
## 55 0.84375 0.700 166.91176
## 56 0.84375 0.725 156.61765
## 57 0.84375 0.750 143.38235
## 58 0.84375 0.775 140.44118
## 59 0.87500 0.775 121.67369
## 60 0.87500 0.800 119.11765
## 61 0.87500 0.825 111.02941
## 62 0.87500 0.850 105.88235
## 63 0.87500 0.875 102.94118
## 64 0.87500 0.900 100.73529
## 65 0.90625 0.900  83.80494
## 66 0.93750 0.900  76.52017
```

```
## 67 0.96875 0.900  71.40277
## 68 1.00000 0.900  68.81397
## 69 1.00000 0.925  47.79412
## 70 1.00000 0.950  44.85294
## 71 1.00000 0.975  36.02941
## 72 1.00000 1.000  32.35294
```

Optimal cutoff using the best negative likelihood ratio:

```
summary(optimal.cutpoints(cerab~cancer, tag.healthy  = 0, methods =
"ValueDLR.Negative", data = data))

##
## Call:
## optimal.cutpoints.formula(X = cerab ~ cancer, tag.healthy = 0,
##     methods = "ValueDLR.Negative", data = data)
##
## Area under the ROC curve (AUC):  0.788 (0.668, 0.908)
##
## CRITERION: ValueDLR.Negative
## Number of optimal cutoffs: 2
##
##                   Estimate
## cutoff       184.5588235
## Se             0.8125000
## Sp             0.3750000
## PPV            0.5098039
## NPV            0.7142857
## DLR.Positive   1.3000000
## DLR.Negative   0.5000000
## FP            25.0000000
## FN             6.0000000
##
##                   Estimate
## cutoff       270.2588020
## Se             0.5000000
## Sp             1.0000000
## PPV            1.0000000
## NPV            0.7142857
## DLR.Positive         Inf
## DLR.Negative   0.5000000
## FP             0.0000000
## FN            16.0000000
```

Based on the above analysis patients who were true positives were considered to have high levels of anti-ceramide antibody, and false negatives were considered low level patients.
